# An Optimized Immunohistochemistry Technique Improves NMO-IgG Detection: Study Comparison with Cell-Based Assays

**DOI:** 10.1371/journal.pone.0079083

**Published:** 2013-11-04

**Authors:** Romana Höftberger, Lidia Sabater, Romain Marignier, Fahmy Aboul-Enein, Raphaël Bernard-Valnet, Helmut Rauschka, Anne Ruiz, Yolanda Blanco, Francesc Graus, Josep Dalmau, Albert Saiz

**Affiliations:** 1 Service of Neurology, Hospital Clínic, Universitat de Barcelona and Institut d´Investigació Biomèdica August Pi i Sunyer (IDIBAPS), Barcelona, Spain; 2 Institute of Neurology, Medical University of Vienna, Vienna, Austria; 3 Team ONCOFLAM, Lyon Neuroscience Research Center INSERM U 1028/CNRS 5292, Lyon, France; 4 Department of Neurology, SMZ-Ost Donauspital, Vienna, Austria; 5 Institució Catalana de Recerca i Estudis Avançats (ICREA), IDIBAPS, Hospital Clínic, Barcelona, Spain; 6 Department of Neurology, University of Pennsylvania, Philadelphia, Pennsylvania, United States of America; Friedrich-Alexander University Erlangen, Germany

## Abstract

Cell-based assays (CBA) have increased the sensitivity of the neuromyelitis optica (NMO)-IgG/aquaporin-4-antibody detection compared to classical tissue-based indirect assays. We describe the sensitivity of an optimized immunohistochemistry (IHC-o) to detect NMO-IgG/aquaporin-4-antibody in comparison with that of two CBA: an in-house (CBA-ih) and a commercial (CBA-c) assay (Euroimmun, Germany). Coded serum from 103 patients with definite NMO and 122 inflammatory controls were studied by IHC-o, CBA-ih, and CBA-c. IHC-o used the same protocol described to detect antibodies against cell surface antigens. CBA-ih used live cells transfected with the aquaporin-4-M23-isoform. The sensitivity of the IHC-o was 74.8% (95% confidence interval [CI] 65-83) and was similar to that of the CBA-ih 75.7% (95% CI 66-84) and the CBA-c 73.8% (95% CI 64-82). The specificity of the three assays was 100% (95% CI 97-100). Interassay concordance was high, 100 of 103 samples were coincident in all techniques. The optimized immunohistochemistry proves to be as sensitive and specific as the cell-based assays. This assay extends the available tools for NMO-IgG/aquaporin-4-antibody detection.

## Introduction

Neuromyelitis optica (NMO) is an inflammatory demyelinating disease of the central nervous system (CNS) characterized by predominant involvement of the optic nerves and spinal cord. For long time, NMO was thought to be a variant of multiple sclerosis (MS), although the prognosis and the response to the therapy was different [1]. The identification of a specific serum autoantibody marker by tissue-based indirect immunofluorescence (IIF), NMO-IgG, that bound to astrocytic membranes and the recognition of the target antigen as the water channel aquaporin-4 (AQP4) [2], led to expand the clinical spectrum of NMO to limited forms of the disease, to define a new set of diagnostic criteria, and to expedite the diagnosis and treatment of the patients [1,3,4,5,6,7,8]. 

Since the initial description of the NMO-IgG/AQP4-antibody, several techniques of detection with different sensitivities and specificities have been reported [9]. In a recent comparative study, IIF was the least and cell-based assay transfected with AQP4 (CBA) the most sensitive assay for NMO-IgG/AQP4-antibody detection [10,11]. In spite of assay refinement, around 20-30% of patients clinically diagnosed with NMO still remain NMO-IgG seronegative [10]. In neuronal autoimmune disorders of the CNS (or autoimmune encephalitis) most of the antibodies were initially identified using IIF or immunohistochemical techniques [12]. These techniques allow the possibility to identify new or coexisting antibodies. We observed that the optimized immunohistochemistry technique (IHC-o) developed for the detection of antibodies against cell surface/synaptic antigens [13], also identified the NMO-IgG pattern, which was easily recognized compared with conventional immunohistochemistry (IHC-c) [7,14]. 

The aim of the current study was to determine the sensitivity and specificity of the IHC-o to detect NMO-IgG/AQP4-antibodies, and compare them with those of conventional tissue-based assays, including IIF and IHC-c, and two CBA, an in-house assay (CBA-ih) with the AQP4-M23 isoform and a commercial assay (CBA-c) [15]. 

## Material and Methods

### Patients

Serum samples from 103 patients with definite NMO according to the revised diagnostic criteria of 2006 [5] (79% female, mean age at sampling 42.1 years, range 7-82 years) and 122 with inflammatory neurological diseases: 101 patients with MS, 30 of them with paired serum and cerebrospinal fluid (83 relapsing and 18 primary progressive MS) fulfilling the McDonalds criteria [16], and 21 with neurological syndromes associated with anti-neuronal antibodies (3 Hu, 2 Ri, 2 Yo, 3 CV2/CRMP5, 2 Ma2, 1 SOX, 3 GAD, 3 LGI1, and 2 CASPR2) were tested by IHC-o, CBA-ih, and CBA-c. The NMO samples were provided by 3 centers: Lyon Neuroscience Research Center, France; Neuroimmunology Group, Hospital Clinic de Barcelona, Spain; and the Department of Neurology, SMZ-Ost Donauspital, Vienna, Austria [17]. Thirty-nine NMO samples have been previously analysed by IIF [6] and other 43 samples by IHC-c [14]. These samples were further re-analyzed by IIF and IHC-c, respectively. Sera were coded before testing and all studies were evaluated by two investigators (RH and AS), blinded to the neurological diagnosis or results of the conventional tissue-based assays.

### Standard Protocol Approvals, Registrations, and Patient Consents

Serum samples used in the study are deposited in the collection of biological samples named "neuroinmunología" registered in the biobank of  Institut d' Investigació Biomèdica August Pi i Sunyer (IDIBAPS), Barcelona, Spain, the biobank Neurobiotec (Hospices Civils de Lyon, France), and SMZost Donauspital, Vienna, Austria (EK11-056VK). Considering that the study was completely anonymous so no sample could be identified to a particular patient, it was accepted to waive the specific written informed consent from the patients or next of kin by the Comitè Ético de Investigación Clínica of Hospital Clínic de Barcelona. Animal handling procedures were approved by the Local Ethics Committee (99/1 University of Barcelona) and the Generalitat de Catalunya (1094/99), in accordance with the Directive 86/609/EU of the European Commission. The study as explained was approved by the Ethical Committee of the Institutional Review Boards of the University of Lyon, Hospital Clínic de Barcelona, and SMZost Donauspital, Vienna.

### Conventional immunohistochemistry technique (IHC-c) and tissue-based indirect immunofluorescence (IIF)

 We used adult male Spraguey Dawley rats that were anaesthesized, sacrificed and perfused with 2% paraformaldehyde. Rat brains were removed, sagittally sectioned and fixed for 4 hours in 2% paraformaldehyde at 4°C. Subsequently, brains were cryoprotected with 20% sucrose for 48h, embedded in freezing medium, and snap frozen in isopentane chilled with liquid nitrogen. Seven micron thick cryostat-cut sections from the cerebellum were defrosted for 20 minutes, washed once in PBS and then incubated with 10% goat serum diluted in 0.03% Triton X-100 (Sigma-Aldrich, St Louis, MO, USA) for 30 minutes. For IHC-c, sections were incubated with patients´sera (1:500, diluted in 10% goat serum in 0.03% Triton-X-100) for 3 hours at 37°C, then they were washed 2x in PBS, labeled with biotinylated goat anti-human IgG (Vector lab) (1:8000, diluted in PBS) for 30 minutes, washed 2x in PBS, and incubated with avidin-biotin peroxidase for 30 minutes at room temperature. The reactivity was developed with diaminobenzidine for 1 to 1.5 minutes (Vector lab). 

For the IIF, we used the same protocol as described above, but with sera diluted 1:50 and sections labeled with an Alexa Fluor secondary antibody against human IgGs (1:500; Molecular Probes, Invitrogen, Eugene, OR, USA) for 2h.

### Optimized immunohistochemistry technique (IHC-o)

We used adult female Wistar rats that were sacrificed in a CO_2_ chamber. Non-perfused rat brains were removed, sagittally sectioned and fixed for 1h in 4% paraformaldehyde at 4°C. Subsequently, brains were cryoprotected with 40% sucrose for 48h, embedded in freezing medium, and snap frozen in isopentane chilled with liquid nitrogen. Seven micron thick cryostat-cut sections were defrosted for 20 minutes, washed once with PBS and then incubated with 0.3% hydrogen peroxide for 15 minutes. After washing 3x with PBS, slides were incubated with 5% goat serum in PBS for 1h, and then labeled with patients´or control sera (1:200, diluted in 5% goat serum) or CSF (1:2) at 4°C overnight. The next day, sections were washed 3x in PBS, labeled with biotinylated goat anti-human IgG (Vector lab) (1:2000, diluted in 5% goat serum) for 2h, washed 3x in PBS, and incubated with avidin-biotin peroxidase for 1h at room temperature. The reactivity was developed with diaminobenzidine for 7 minutes (Vector lab).

### Cell-based assay

Two CBA were performed. A commercial (CBA-c) following the manufacturer´s instructions (Euroimmun, Luebeck, Germany) [15], and an in-house CBA (CBA-ih) with cells transfected with the M23 isoform of AQP4 (the clone was a gift from Dr. R. Marignier). Briefly, thirty-six hours after transfecting HEK293 cells with the AQP4-M23 isoform, live cells were incubated at room temperature with centrifuged serum 1:20 and CSF 1:2 (diluted in DMEM with HEPES and 1% bovine serum albumine) for 30 minutes. After removing the media and washing with DMEM with HEPES and 1% BSA, HEK cells were fixed with 1% paraformaldehyde for 15 minutes and permeabelized with 0.3% Triton X-100 (Sigma-Aldrich, St Louis, MO, USA). HEK cells were then immunolabeled with a rabbit polyclonal anti-AQP4 antibody (1:500; Sigma-Aldrich) for 1h at room temperature, followed by the corresponding Alexa Fluor secondary antibodies against human and rabbit IgGs (1:1000; Molecular Probes, Invitrogen, Eugene, OR, USA). 

### Statistical analysis

The receiver operating characteristic curve (ROC) analysis was used for the assessment of the diagnostic accuracy of these tests. The degree of concordance between two assays was assessed by the kappa statistic, which measures agreement beyond chance. Kappa (κ) equals 1.0 for perfect agreement and values above 0.8 are considered to represent very good agreements. We used SPSS software version 18.0 for all calculations. 

## Results

### Staining pattern of NMO-IgG/AQP4-antibodies with the optimized immunohistochemistry technique

**Figure 1:**
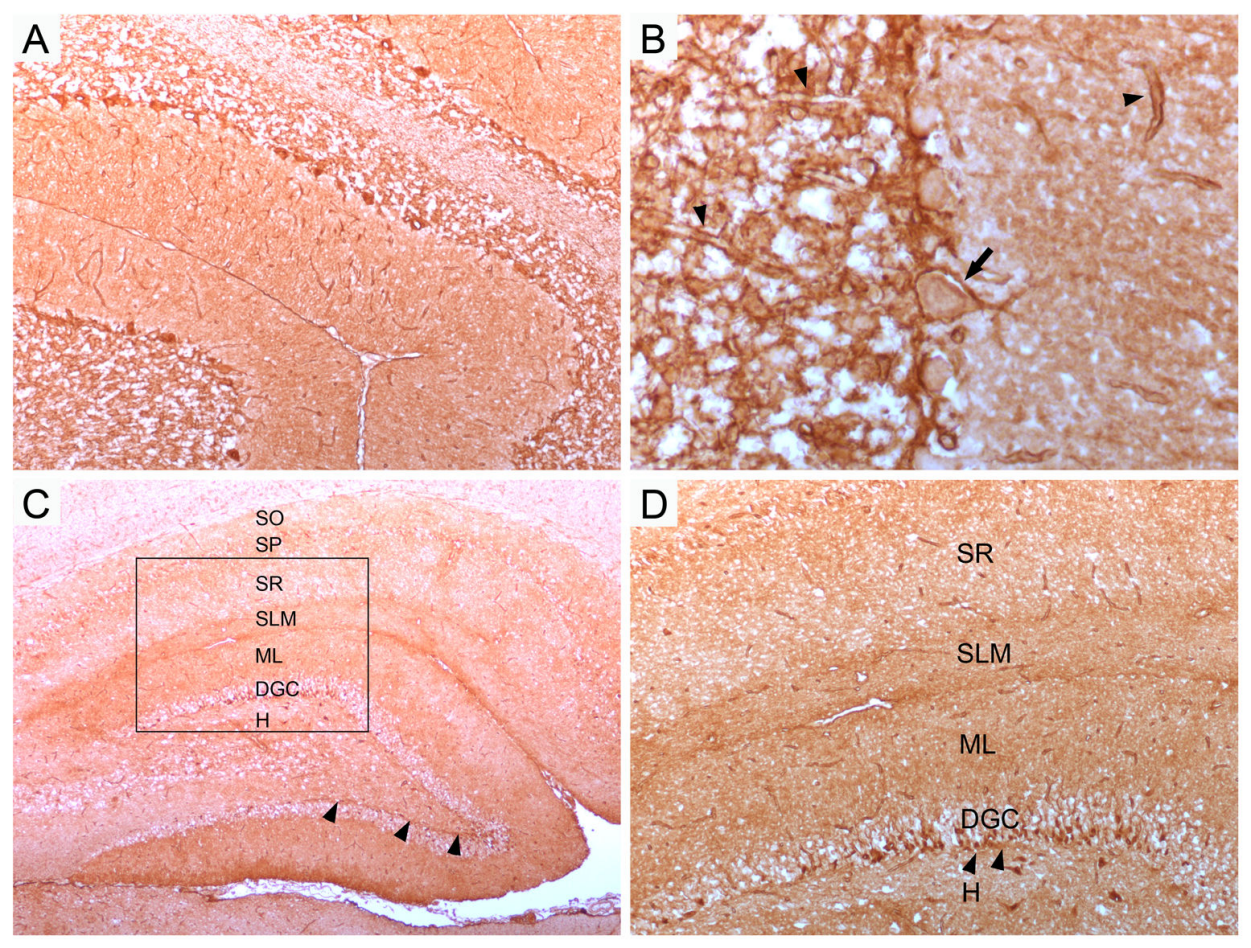
Staining pattern of a patients´ serum with NMO-IgG/AQP4-antibodies in rat brain. Patients´serum shows extensive labeling of the granular layer of the cerebellum (A). Note the reticular staining pattern, forming basket-shaped processes around cell bodies of Purkinje cells (arrow), and the glia limitans perivascularis (arrow heads) (B). Immunoreactivity of hippocampus shows laminar specificity with strongest staining in the stratum lacunosum moleculare (SLM), molecular layer of the dentate gyrus (ML) and a thin layer of AQP4-positivity in the subgranular zone (arrow heads) (C, D; rectangle in C enlarged in D). SO, stratum oriens; SP, stratum pyramidale; SR, stratum radiatum; DGC, dentate granule cell layer; H, hilus. Magnification: A, D x100; B, x400; C, x40; .

The IHC-o specifically detects antibodies targeting AQP4 on the astroglial cell surface. The staining pattern on rat brain sections is characterized by 1) reticular labeling of glial processes in the granular layer of the cerebellum (Figure 1A), forming basket-shaped branches around cell bodies of Purkinje cells (Figure 1B arrow) 2) labeling of the glia limitans perivascularis throughout the whole brain (Figure 1B arrowheads), and 3) fine process staining in the neuropil of the hippocampus with denser immunoreactivity in the stratum lacunosum moleculare, the molecular layer of the dentate gyrus and a thin layer of AQP4-positivity in the subgranular zone (Figure 1C, D arrowheads). Patients with low antibody titer may only show staining of the granular layer of the cerebellum and labeling of the glia limitans perivascularis. 

### Sensitivity and specificity of the NMO-IgG/AQP4-antibody assays

The sensitivity of the IHC-o was 75% (77 of 103), the CBA-ih 76% (78 of 103), and the CBA-c 74% (76 of 103) (Table 1). Interassay concordance overall was high, 100 of 103 were coincident in all techniques (Table 2). The specificity of the three assays was 100%. The concordance kappa value between CBA-ih and IHC-o was 0.99 (95% confidence interval [CI] 0.987-0.992), between CBA-ih and CBA-c 0.980 (0.975-0.985) and between IHC-o and CBA-c 0.970 (0.962-0.977) (p <0.0001 for all values). The interobserver variability for the IHC-o was low and only discordant in two samples, the concordance kappa value between the two investigators was 0.980 (95% CI 0.974-0.985). The IIF and IHC-c analysis showed a complete agreement with the previous results. The IHC-o detected 6 more positive cases than the IIF (24 of 39; 61.5% vs 46%) and 5 more than the IHC-c (33 of 43; 77% vs 65%) (Table 2). 

**Table 1:** Sensitivity and specificity of the NMO-IgG/AQP4-antibody assays.

	**NMO** n=103	**Control** n=122	**Sensitivity** (95% CI)	**Specificity** (95% CI)	**ROC - AUC** (95% CI)	**Pos. LR** (95% CI)	**Negative LR** (95% CI)
**CBA-ih**	78	0	75.7 (66.3-83.6)	100 (96.9-100)	0.879 (0.83-0.93)	∞ (n.a)	0.24 (0.17-0.34)
**IHC-o**	77	0	74.8 (65.2-82.8)	100 (96.9-100)	0.874 (0.82-0.93)	∞ (n.a)	0.25 (0.18-0.35)
**CBA-c**	76	0	73.8 (64.2-82)	100 (96.9-100)	0.869 (0.82-0.92)	∞ (n.a)	0.26 (0.19-0.36)

CBA-ih=in-house cell-based assay. IHC-o=optimized immunohistochemical technique. CBA-c=commercial cell-based assay. NMO=neuromyelitis optica. ROC-AUC=receiver operating characteristic curve-area under the curve. CI=confidence intervals. Pos. LR=positive likelihood ratio. Negative LR=negative likelihood ratio. n.a.=not applicable.

**Table 2:** Concordance of the different assays.

**NMO cases France** (n=39)	**IHC-o**	**CBA-ih**	**CBA-c**	**IIF**	**NMO cases Spain**(n=43)	**IHC- o**	**CBA- ih**	**CBA-c**	**IHC-c**	**NMO cases Austria** (n=21)	**IHC- o**	**CBA- ih**	**CBA- c**
	+	+	+	+		+	+	+	+		+	+	+
	+	+	+	+		+	+	+	+		+	+	+
	+	+	+	+		+	+	+	+		+	+	+
	+	+	+	+		+	+	+	+		+	+	+
	+	+	+	+		+	+	+	+		+	+	+
	+	+	+	+		+	+	+	+		+	+	+
	+	+	+	+		+	+	+	+		+	+	+
	+	+	+	+		+	+	+	+		+	+	+
	+	+	+	+		+	+	+	+		+	+	+
	+	+	+	+		+	+	+	+		+	+	+
	+	+	+	+		+	+	+	+		+	+	+
	+	+	+	+		+	+	+	+		+	+	+
	+	+	+	+		+	+	+	+		+	+	+
	+	+	+	+		+	+	+	+		+	+	+
	+	+	+	+		+	+	+	+		+	+	+
	+	+	+	+		+	+	+	+		+	+	+
	+	+	+	+		+	+	+	+		+	+	+
	+	+	+	+		+	+	+	+		+	+	+
	+	+	+	-		+	+	+	+		+	+	+
	+	+	+	-		+	+	+	+		+	+	+
	+	+	+	-		+	+	+	+		-	+	+
	+	+	+	-		+	+	+	+				
	+	+	-	-		+	+	+	+				
	+	+	-	-		+	+	+	+				
	-	-	-	-		+	+	+	+				
	-	-	-	-		+	+	+	+				
	-	-	-	-		+	+	+	+				
	-	-	-	-		+	+	+	+				
	-	-	-	-		+	+	+	-				
	-	-	-	-		+	+	+	-				
	-	-	-	-		+	+	+	-				
	-	-	-	-		+	+	+	-				
	-	-	-	-		+	+	+	-				
	-	-	-	-		-	-	-	-				
	-	-	-	-		-	-	-	-				
	-	-	-	-		-	-	-	-				
	-	-	-	-		-	-	-	-				
	-	-	-	-		-	-	-	-				
	-	-	-	-		-	-	-	-				
						-	-	-	-				
						-	-	-	-				
						-	-	-	-				
						-	-	-	-				
**Pat.**	24/ 39	24/ 39	22/ 39	18/ 39	**Pat.**	33/ 43	33/ 43	33/ 43	28/ 43	**Pat.**	20/ 21	21/ 21	21/ 21
**Co.**	0/ 122	0/ 122	0/ 122										

NMO=neuromyelitis optica. IHC-o=optimized immunohistochemical technique. CBA-ih=in-house cell-based assay. CBA-c=commercial cell-based assay. IIF=indirect immunofluorescence. Pat=patients. Co=controls.

## Discussion

The identification of NMO-IgG/AQP4-antibody is essential to establish an early diagnosis of NMO and to promptly initiate the appropriate immunotherapy. Several methods for the detection of NMO-IgG/AQP4-antibody are available, which differ in their sensitivities and specificities [9]. In a recent multicenter comparative study, assays based on binding of IgG to cells transfected with AQP4 proved to be the most and IIF the least sensitive method of detection [10]. However, screening for NMO-IgG/AQP4-antibody by tissue-based assays is still recommended as it is cheap and can demonstrate different or new antibodies associated with NMO disease spectrum, while the CBA only demonstrates the antigen of interest overexpressed in the cells.

In this blinded study we show that the IHC-o commonly used to identify recognizable staining patterns of antibodies against cell surface/synaptic proteins also detects NMO-IgG/AQP4-antibodies with a higher sensitivity compared to the conventional tissue-based assays, without losing specificity. In fact, with this method our previously reported sensitivity of 65% increased to 77% [14]. This high sensitivity and specificity of IHC-o compares well with that of CBA; the agreement with results obtained with the CBA-ih was very high and only one sample was not identified with the IHC-o. It is important to bear in mind that the aim of the current study was to compare the sensitivity and specificity of different methods of NMO-IgG/AQP4-antibody detection but not to address their value in the diagnosis of NMO/NMO spectrum disorders, which is limited by bias in the selection of the samples. 

The IHC-o identifies a characteristic staining pattern that corresponds well to the anatomical distribution of AQP4 in the brain [18]. The pattern is well recognized despite the presence of other antibodies (e.g. NMDAR antibodies); for example, we did not find false positive results despite the large representation of samples with known neuronal antibodies included as controls. As expected, the sensitivity of the commercial CBA-c was also very high.

We realize that the sensitivity of the IHC-o may depend on laboratory expertise. In some patients with very low titer of antibodies, the IHC-o may show only weak staining of the granular cell layer of the cerebellum with lower immunostaining of the glia limitans perivascularis. In such cases and especially in clinically well-selected patients, we suggest complementing this study with the commercial CBA-c. In fact, none of the patients of the current study was misdiagnosed when combining both techniques.

The number of assays for NMO-IgG/AQP4-antibody detection is increasing, but seronegative NMO-IgG/AQP4-antibody status remains a challenge. Myelin-oligodendrocyte glycoprotein antibodies have been reported in a few NMO-IgG/AQP4-antibody seronegative patients with NMO [19,20,21]; it is reasonable to expect that novel antibodies will be identified in other NMO seronegative patients. Detection of some of these antibodies will be facilitated by the immunohistochemistry reported here. For example, in the past 12 months we tested over 300 samples from patients in whom NMO was suspected. In one patient with isolated longitudinally extensive transverse myelitis, the IHC-o identified LGI1 antibodies instead of NMO-IgG; the identity of LGI1 was subsequently confirmed with the appropriate CBA. In another patient, a neuropil pattern of brain immunostaining was detected, and an antibody against a neuronal cell-surface antigen was subsequently confirmed with immunostaining of live hippocampal neurons. Altogether, these data support the additional value of using tissue-based assays.

In conclusion, this study shows that the indicated IHC-o is sensitive to detect NMO-IgG/AQP4-antibody and the findings compare well with CBA. This type of IHC expands the available tools for NMO-IgG/AQP4-antibody detection, and offers a technique that has the advantage to detect other cell surface antibodies that would be missed if the NMO-IgG/AQP4-antibody detection is directly done by CBA. 
